# P-glycoprotein is expressed and causes resistance to chemotherapy in EBV-positive T-cell lymphoproliferative diseases

**DOI:** 10.1002/cam4.494

**Published:** 2015-07-08

**Authors:** Mayumi Yoshimori, Honami Takada, Ken-Ichi Imadome, Morito Kurata, Kouhei Yamamoto, Takatoshi Koyama, Norio Shimizu, Shigeyoshi Fujiwara, Osamu Miura, Ayako Arai

**Affiliations:** 1 Department of Hematology, Graduate School of Medical and Dental Sciences, Tokyo Medical and Dental UniversityTokyo, Japan; 2 Department of Laboratory Molecular Genetics of Hematology, Graduate School of Health Care Sciences, Tokyo Medical and Dental UniversityTokyo, Japan; 3 Division of Advanced Medicine for Virus Infections, National Research Institute for Child Health and DevelopmentTokyo, Japan; 4 Department of Comprehensive Pathology, Graduate School of Medical and Dental Sciences, Tokyo Medical and Dental UniversityTokyo, Japan; 5 Division of Medical Science, Department of Virology, Medical Research Institute, Tokyo Medical and Dental UniversityTokyo, Japan; 6 Department of Allergy and Clinical Immunology, National Research Institute for Child Health and DevelopmentTokyo, Japan

**Keywords:** Drug resistance, EBV, lymphoproliferative disorders, P-glycoprotein, T-cell lymphoma

## Abstract

Epstein–Barr virus-positive T-cell lymphoproliferative diseases (EBV-T-LPDs) are rare lymphomas with poor prognosis. Although chemotherapeutic strategies such as CHOP have been often selected, they have exhibited only limited efficacy. To clarify the mechanism of chemoresistance, we examined P-glycoprotein (P-gp) expression. P-gp acts as an energy-dependent efflux pump that excretes drugs from the cytoplasm, resulting in low-intracellular drug concentrations and poor sensitivity to chemotherapy. We examined P-gp expression in EBV-positive cells by immunohistochemistry staining in three patients of EBV-T-LPDs and the expression was detected in all patients. We also examined *mdr1* mRNA expression by reverse-transcriptase polymerase-chain reaction (RT-PCR) in EBV-positive tumor cells from these patients and additional three patients. The expression was detected in all examined patients. In five EBV-T-LPDs patients, P-gp function was detected by Rhodamine-123 efflux assay in these cells. The efflux was inhibited by treatment with a P-gp inhibitor, cyclosporine A (CsA). We also examined and detected P-gp expression in EBV-positive T-cell lines SNT8 and SNT16 established from EBV-T-LPDs patients, by RT-PCR and western blotting. The function was also detected by Rhodamine-123 efflux in these cell lines. Inhibition and knock down of P-gp by CsA and siRNA, respectively, enhanced etoposide- and doxorubicin-induced cell death in the EBV-positive T-cell lines. Finally, we infected the T-cell line MOLT4 with EBV, and found that *mdr1* mRNA expression and Rhodamine 123 efflux were upregulated after infection. These results indicated that enhanced P-gp expression contributed to the chemoresistance of EBV-T-LPDs.

## Introduction

Epstein–Barr virus (EBV) can infect not only B cells but also rarely T or natural killer (NK) cells in EBV-positive T/NK-cell neoplasms, such as extranodal NK/T-cell lymphoma (ENKL), aggressive NK-cell leukemia, some peripheral T-cell lymphomas not otherwise specified (PTCL-NOS), and EBV-positive T-cell lymphoproliferative diseases (EBV-T-LPDs). EBV-T-LPDs were originally reported as a disease of children and young adults with sustained infectious mononucleosis-like symptoms and were named as chronic active EBV infection (CAEBV) 1. In the late 1980s, however, Jones and colleagues reported that some CAEBV cases were accompanied by EBV-positive clonally proliferating T cells 2. Following reports supported the findings and demonstrated that the disorders were progressive with a poor prognosis. In the WHO classification revised in 2008, therefore, the disorders were named EBV-T-LPDs of childhood and classified under peripheral T/NK-cell neoplasms 3.

EBV-T-LPDs are accompanied by severe inflammatory symptoms such as fever, liver dysfunction, and hemophagocytic lymphohistiocytosis. Some cases reveal characteristic skin lesions such as hypersensitivity to mosquito bites (HMB) or hydroavacciniforme (HV). Most cases have no solid tumor, however, have EBV-positive and clonally proliferating T cells in the peripheral blood. They are rapidly progressive and the outcomes are quite poor 4. Koyama and colleagues reported the effects of sequential chemotherapy consisting of immunochemotherapy: cyclosporine A (CsA), etoposide, and prednisolone followed by CHOP and Capizzi 5. This strategy has been widely used as an induction therapy of EBV-T-LPDs. However, the efficacy for the disorders was limited and EBV-positive cells could not be eradicated in most cases 6. Although stem cell transplantation may be curative 7,8, no definitively curative chemotherapy regimen has been identified for EBV-T-LPDs to date.

P-glycoprotein (P-gp) is a membrane transporter and a product of the multiple drug resistance 1 (*mdr1*) gene 9. It acts as an energy-dependent efflux pump that excretes drugs from the cytoplasm, resulting in low-intracellular drug concentrations and poor sensitivity to chemotherapy 10,11. It was previously reported that the tumor cells of ENKL, one type of EBV-positive NK -cell neoplasms, expressed P-gp, resulting in high resistance to chemotherapy 12. Based on the report, combination chemotherapy consisting of P-gp-independent reagents for ENKL have been suggested and the effects have been reported recently.

In this report, we investigated the expression and function of P-gp in cells derived from EBV-T-LPDs patients to clarify the mechanism of chemoresistance of the disease.

## Materials and Methods

### Cells and reagents

We used the EBV-positive T- or NK cell lines SNT8, 16, and SNK6. SNT8 and SNT16 were established from EBV-T-LPDs and SNK6 was from ENKL 13. They were maintained in Roswell Park Memorial Institute medium 1640 medium containing 10% fetal calf serum (10% FCS–RPMI) and human interleukin-2 (IL-2) as described previously 13. An EBV-negative B-cell line, MD901, was established from cells in the pleural effusion of a patient with “variant type” Burkitt's lymphoma 14, which responded to CHOP regimen, and maintained in 10% FCS–RPMI. The EBV-negative T-cell lines Jurkat, MOLT4 and EBV-positive B-cell lines, Raji, HS-sultan were cultured in 10% FCS–RPMI 15. IL-2 was purchased from R&D systems (Abington, UK). Etoposide, doxorubicin, and CsA were purchased from Wako Pure Chemical Industries (Osaka, Japan). L-asparaginase (L-asp) was kindly provided by Kyowa-Hakko Kirin Co. Ltd (Tokyo, Japan). Rhodamine-123 was purchased from Sigma-Aldrich (St. Louis, MO).

### Diagnosis of EBV-T-LPDs

EBV-T-LPDs were diagnosed based on the criteria as suggested by Kimura and colleagues 16. Briefly, (1) clinical findings described in the previous reports, presence of characteristic clinical findings such as persistent IM-like symptoms, HMB, HV, (2) high-EBV load detected in peripheral blood mononuclear cells (PBMCs) by quantitative polymerase chain reaction (PCR) (>10^2.5^ copies/*μ*g of EBV DNA), and (3) EBV infection on T cells.

### Detection and isolation of EBV-infected cells in EBV-T-LPDs patients

The detection and isolation of infected cells were performed as described previously 17. Briefly, PBMCs from patients were isolated by density gradient centrifugation using Separate-L (Muto Pure Chemical, Tokyo, Japan) and sorted into CD19-, CD4-, CD8-, or CD56-positive fractions by using antibody-conjugated magnetic beads (IMag Human CD4, 8, and 56 Particles-DM; BD Biosciences, Sparks, MD). EBV DNA levels in each fraction were then measured by real-time reverse-transcriptase PCR (RT-PCR) using the TaqMan system (Applied Biosystems, Foster City, CA) 18. The fraction with the highest titer was assumed to be with the fraction containing the infected cells. For the assays, we isolated EBV-infected cells from PBMCs using magnetic beads-conjugated antibodies targeting the surface markers of the infected cells. The clonality of the infected cells was examined by Southern blotting.

### Antibodies

Anti-P-gp and anti-*α*-tubulin antibodies for western blotting were purchased from Abcam (Cambridge, MA) and Santa Cruz Biotechnology (Santa Cruz, CA), respectively.

### Western blotting

The assay was performed as described previously 19. Briefly, after washing with phosphate buffered saline, cells were lysed in SIP buffer (50 mmol/L Tris–HCl pH 7.5, 5 mmol/L ethylenediaminetetraacetic acid, 100 mmol/L NaCl, 50 mmol/L NaF, 1 mmol/L Na_3_VO_4_, 40 mmol/L *β*-glycerophosphate, and 1% Triton X-100). The resulting lysate was centrifuged at 170g for 10 min, and the supernatant was collected and subjected to western blotting.

### Immunohistochemistry

The 4 *μ*m thick paraffin-embedded formalin-fixed tissue sections were de-paraffinized, and heat-based antigen retrieval was performed in 0.1 mol/L citrate buffer (pH 6.0). Endogeneous peroxidase activity was inhibited using hydrogen peroxide. The primary antibody for P-gp (ab98322) was purchased from Abcam (Cambridge, MA). The detection system was the streptavidin-biotin-peroxidase complex technique (ABC kit; Vector Laboratories, Burlingame, CA) with diaminobenzidine (DAB; Nichirei Bioscience, Tokyo, Japan) as the chromogen. In situ hybridization (ISH) of Epstein–Barr virus-encoded small RNA (EBER) was performed for detection of EBV in tissue sections by EBV (EBER) PNA Probe/Fluorescein (DAKO, Carpinteria, CA) and second antibody for Fluorescein (Dako, Glostrup, Denmark). For double staining for EBER and P-gp, anti-P-gp staining was performed with nickel DAB as chromogen (Vector Laboratories, Burlingame, CA), followed by the ISH.

### RT-PCR for *mdr1* expression

mRNA was measured by real-time RT-PCR using TaqMan system. Oligonucleotides (as specific primers) and TaqMan probes for the *mdr1* and glyceraldehyde-3-phosphate dehydrogenase (*GAPDH*) mRNA were purchased from Applied Biosystems (Foster, CA). Data on the quantity of RNA for *mdr1* were normalized using the data for *GAPDH* in each sample. The data were analyzed by the 2(-Delta Delta C (T)) Method 20.

### Rhodamine-123 efflux assay

The assay was performed as described previously 21,22. Briefly, cells were washed once and resuspended in 10% FCS–RPMI with 500 ng/mL Rhodamine-123. They were incubated for 30 min at 37°C. After two washes, they were allowed to efflux the dye in dye-free 10% FCS–RPMI for 2 h at 37°C or 4°C. The assay was also performed at 37°C with 2 *μ*mol/L CsA, a potent inhibitor of P-gp. After efflux, cells were analyzed using a FACS Calibur flow cytometer (Becton, Dickinson and Company, Franklin Lakes, NJ).

### Detection of cell viability

Cell viability was examined by trypan blue staining.

### Knock down of *mdr1* by siRNA

siRNA-*mdr1* and control siRNA were from Santa Cruz Biotechnology (Santa Cruz, CA). For the introduction of siRNA into SNT8 and SNT16 cells, 5 × 10^6^ cells were transfected with 6 *μ*g of siRNA according to the manufacturer's instructions. The transfected cells were cultured for 48 h and used for the examination.

### *In vitro* EBV infection assay

*In vitro* EBV infection assay was performed as described previously 15,23. Briefly, EBV was prepared from culture medium of B95-8 cells, and then concentrated (200-fold) in RPMI medium 1640 supplemented with 10% FCS. The virus suspension was filtered (0.45 *μ*m) and the recipient cells (2 × 10^6^–1 × 10^7^) were incubated in 1 or 5 mL of the suspension for 1 h, and then rinsed twice with culture medium (10% RPMI). For inactivation of the EBV genome, 1 mL of virus suspension in a 100-mm dish was irradiated with UV (254 nm) at 1 Jμ/cm^2^ using a FUNA-UV-LINKER FS-800 (Funakoshi, Tokyo, Japan). Infection was verified by EBV–DNA quantitation, and immune fluorescence staining of EBV nuclear antigen (EBNA) staining of the cells as described using Polyclonal Rabbit Anti-Human C3c Complement/FITC antibody (Dako, Glostrup, Denmark) 24.

### Ethical statement

The study complied with the principles of the Declaration of Helsinki and was approved by the ethical committee of Tokyo Medical and Dental University. Written informed consent was obtained from all patients.

## Results

### P-gp expression was detected in the EBV-positive T cells of EBV-T-LPDs patients

**Figure 1:**
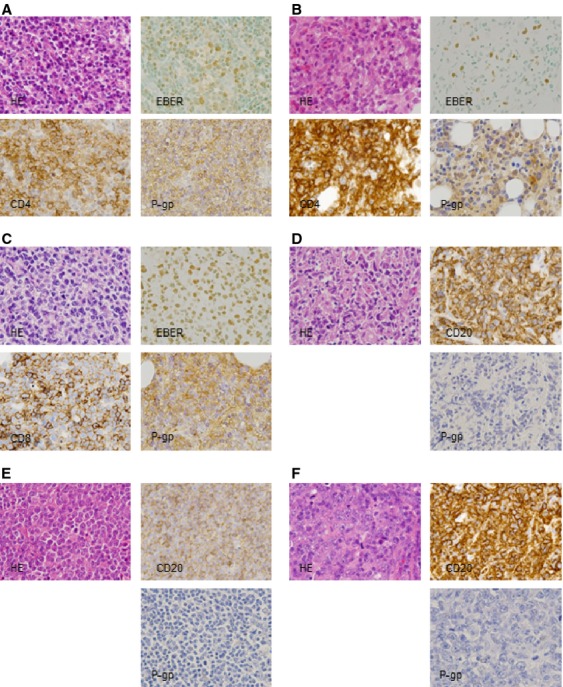
P-gp expression in a EBV-T-LPDs patients was examined by immunochemical staining for histopathologic specimen. (A) CD4-1, (B) CD4-2, (C) CD8-1, (D) DLBCL-1, (E) DLBCL-2, (F) DLBCL-3. Hematoxylin and eosin staining showed diffuse infiltration of lymphocytes. Most cells were positive for CD4 in (A and B), or CD8 in (C). In situ hybridization of EBER demonstrated that most infiltrating cells were positive for EBER. Immunochemical staining with anti-P-gp antibody showed that infiltrating cells were positive for P-gp. In (D–F), Most infiltrating cells were positive for CD20. Immunochemical staining with anti-P-gp antibody showed that infiltrating cells were negative for P-gp. Original magnification was 1000×. EBV-T-LPDs, Epstein–Barr virus-positive T-cell lymphoproliferative diseases; DLBCL, diffuse large B-cell lymphoma; EBER, Epstein–Barr virus-encoded small RNA; P-gp, P-glycoprotein.

First of all, we examined and confirmed P-gp protein expression in the tissue lesion in EBV-T-LPDs by immunohistochemical staining. The tissue samples were obtained from three patients, CD4-1, CD4-2, and CD8-1. The patients were diagnosed according to the previously described diagnostic criteria. Their clinical information was shown in Table1. Clonal proliferation of EBV-infected cells was detected in all cases. Biopsy was performed as diagnostic procedure for the lesions and infiltration had been found. We also investigated the specimen from three EBV-negative diffuse large B-cell lymphoma (DLBCL) patients, whose disease responded to CHOP regimen. Their clinical information was shown in Table S1. As shown in Figure1A–C, most infiltrating cells were positive for CD4 or CD8, and EBER-positive cells were detected in them. In addition, most cells were positive for P-gp. These results indicated that infiltrating EBV-positive T cells expressed P-gp. On the other hand, infiltrating cells in DLBCL patients were negative for P-gp (Fig.1D–F).

**Table 1:** EBV-T-LPDs patients and their EBV-infected cells in the peripheral blood subjected to the assay

Case	Gender	Age	Infected cell	Clinical findings	Clonality of the infected cells	EBV DNA load of each fraction of PBMCs (copies/*μ*gDNA)						
						Total	CD4	CD8	CD56	CD14	CD19	Others
CD4-1	F	25	CD4	HMB	Mono	7.0 × 10^4^	2.2 × 10^5^	ND	ND	ND	ND	ND
CD4-2	M	28	CD4	HLH	Mono	2.3 × 10^4^	9.2 × 10^4^	ND	ND	ND	ND	2.3 × 10^3^
CD4-3	M	41	CD4	sCAEBV	Mono	1.7 × 10^3^	1.7 × 10^4^	ND	ND	ND	1.0 × 10^4^	ND
CD8-1	F	21	CD8	sCAEBV	Mono	1.8 × 10^3^	ND	6.0 × 10^2^	ND	ND	ND	ND
CD8-2	F	28	CD8	HMB, HLH	Mono	4.9 × 10^4^	1.5 × 10^5^	ND	ND	ND	ND	ND
CD8-3	M	28	CD8	sCAEBV	Mono	1.3 × 10^5^	1.2 × 10^2^	2.0 × 10^6^	1.7 × 10^3^	ND	2.0 × 10^4^	ND
CD8-4	F	64	CD8	sCAEBV	Mono	2.6 × 10^5^	ND	1.2 × 10^6^	ND	ND	4.6 × 10^5^	ND

EBV-T-LPDs, Epstein–Barr virus-positive T-cell lymphoproliferative diseases; PBMCs, peripheral blood mononuclear cells; M, male; F, female; HMB, hypersensitivity to mosquito bites; HLH, hemophagocytic lymphohistiocytosis; sCAEBV, systemic chronic active Epstein–Barr virus infection; Mono, monoclonal; ND, not detected.

**Figure 2:**
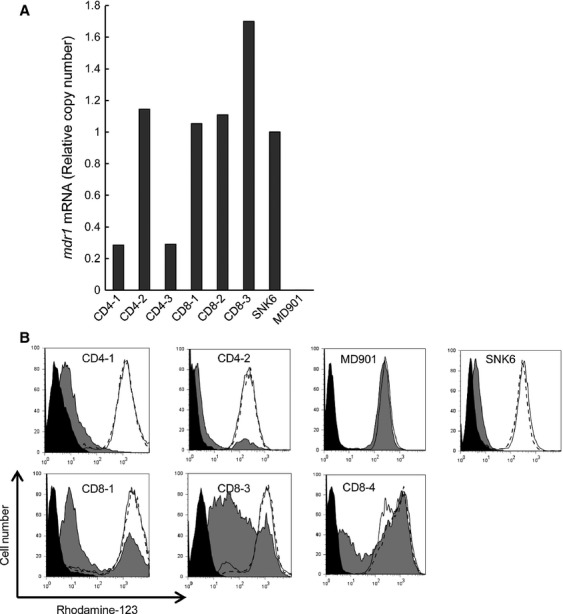
*mdr1* RNA expression in EBV-T-LPDs patient cells. (A) *mdr1* RNA expression in EBV-positive cell fractions of EBV-T-LPDs patients was examined by quantitative RT-PCR assay. Transcripts of *mdr1* and *GAPDH* of each patient were quantitated by real-time RT-PCR. SNK6 and MD901 were examined as a positive and negative control, respectively. Relative copy number was obtained by normalizing the *mdr1* transcripts to those of *GAPDH*. (B) Function of P-gp was examined by Rhodamine-123 efflux assay in EBV-positive cells of EBV-T-LPDs patients. Cells were incubated with 500 ng/mL of Rhodamine-123 for 30 min at 37°C, then allowed to efflux the dye in dye-free 10% FCS-RPMI for 2 h at 37°C (gray, shaded histogram) or at 4°C (open histogram). The assay was also performed at 37°C with 2 *μ*mol/L of CsA, a potent inhibitor of P-gp (open histogram with dot line). After efflux, cells were analyzed using flow cytometer. The untreated cells were presented in black, shaded histogram. EBV-T-LPDs, Epstein–Barr virus-positive T-cell lymphoproliferative diseases; RT-PCR, reverse-transcriptase polymerase chain reaction; *GAPDH*, glyceraldehyde-3-phosphate dehydrogenase; P-gp, P-glycoprotein; FCS, fetal calf serum; CsA, cyclosporine A.

**Figure 3:**
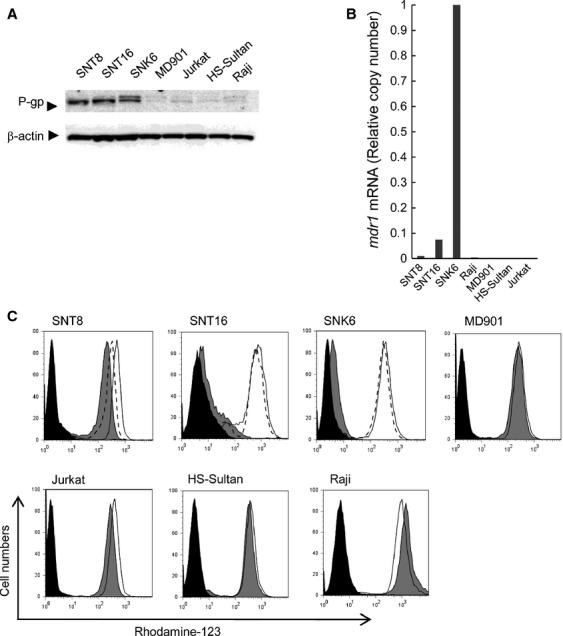
P-gp expression and function in EBV-T-LPDs cell lines. (A) P-gp expression in EBV-T-LPDs cell lines was examined by western blotting. Cells were lysed and subjected to the analysis with anti-P-gp (the upper panel), and *β*-actin (the lower panel) antibodies. (B) *mdr1* RNA expression in the cell line was examined by quantitative RT-PCR assay. Transcripts of *mdr1* and *GAPDH* of each cell line were quantitated by real-time RT-PCR. Relative copy number was obtained by normalizing the *mdr1* transcripts to those of *GAPDH*. (C) Function of P-gp was examined by Rhodamine-123 efflux assay in the cell line. Cells were incubated with 500 ng/mL of Rhodamine-123 for 30 min at 37°C, then allowed to efflux the dye in dye-free 10% FCS-RPMI for 2 h at 37°C (gray, shaded histogram) or at 4°C (open histogram). The assay was also performed at 37°C with 2 *μ*mol/L of CsA, a potent inhibitor of P-gp (open histogram with dot line). After efflux, cells were analyzed using flow cytometer. The untreated cells were presented in black, shaded histogram. P-gp, P-glycoprotein; EBV-T-LPDs, Epstein–Barr virus-positive T-cell lymphoproliferative diseases; RT-PCR, reverse-transcriptase polymerase chain reaction; *GAPDH*, glyceraldehyde-3-phosphate dehydrogenase; FCS, fetal calf serum; CsA, cyclosporine A.

Next, we investigated P-gp gene expression in EBV-positive T- or NK cells from EBV-T-LPDs by RT-PCR. Six EBV-T-LPDs patients were investigated. Their clinical information was also shown in Table1. In EBV-T-LPDs, EBV-positive cells were detected in CD4- or CD8-positive fraction of PBMCs and the fraction was isolated using antibody-conjugated magnetic beads. RNA was extracted immediately from freshly isolated EBV-positive cell fractions and was used for quantitative PCR. As shown in Figure2A, the expression of *mdr1*, which encodes P-gp, was greater in EBV-positive T cells from all of the examined EBV-T-LPDs patients than in EBV-negative cell line MD901, which was established from a patient with EBV-negative B-cell lymphoma responded to CHOP (Fig.3A) 14. An EBV-positive NK cell lines SNK6 which had been established from an ENKL patient was used as a positive control, because the tumor cells from ENKL express P-gp 12. Next, we examined the function of P-gp in EBV-positive cell fractions by the Rhodamine efflux assay. Cells could be used for the assay from five patients shown in Table1. Efflux was detected and markedly inhibited by treatment with a P-gp inhibitor, CsA, in all examined patients (Fig.2B). These results indicated that the EBV-positive T cells from EBV-T-LPDs patients had functional P-gp expression.

### P-gp expression was detected in EBV-T-LPDs cell lines

Next, we investigated P-gp expression in EBV-T-LPDs cell lines. Two EBV-positive T-cell lines, SNT8 and SNT16 had been established from EBV-T-LPDs patients 12. P-gp expression was examined by western blotting and was detected more clearly in these cell lines than in the control cell line MD901 (Fig.3A). Two bands of SNK6 were considered to be splicing variants. The expression was also negative in Jurkat and EBV-positive B-cell lines, HS-sultan and Ramos cells. Quantitative RT-PCR also demonstrated the expression of *mdr1* in SNT8 and SNT16 cells, whereas it was barely expressed in the MD901, Jurkat, and in EBV-positive B-cell lines (Fig.3B). In accordance with these results, functional P-gp expression was detected in these cells. As shown in Figure3C, the efflux of Rhodamine-123, which was excreted from the cytoplasm by P-gp, was detected in SNT8, SNT16, and SNK6 cells but not or faint in MD901, Jurkat, and in EBV-positive B-cell lines. These results indicated that the EBV-T-LPDs cell lines had functional P-gp expression.

### Suppression of P-gp enhanced etoposide- and doxorubicin-induced cell death in EBV-T-LPDs cells

**Figure 4:**
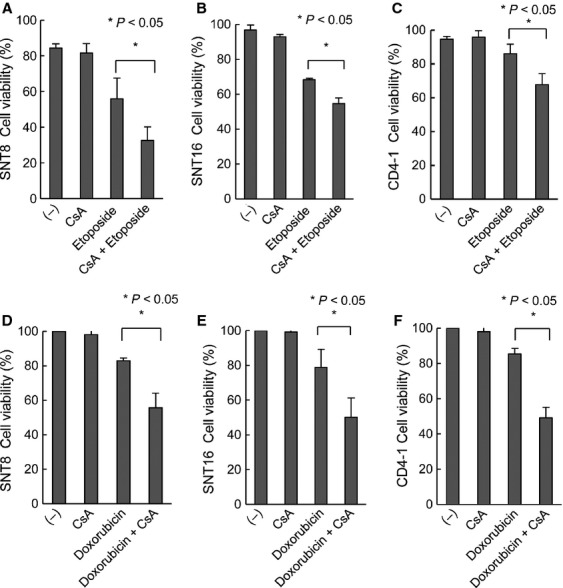
The effects of P-glycoprotein inhibitor, cyclosporine A, on etoposide- and doxorubicin-induced cell death in EBV-T-LPDs cells. (A and B) EBV-T-LPDs cell lines, SNT8 (A) and SNT16 (B) were cultured with 2 *μ*mol/L of cyclosporine A with or without 2 *μ*mol/L of etoposide as indicated for 24 h. Cell viability of each cell line was examined by trypan blue staining. The graph chart represents the mean ± SD of three independent experiments. (C) PBMCs from EBV-T-LPDs patients (case CD4-1) were cultured in 10% FCS–RPMI containing IL-2 with 1 *μ*mol/L of cyclosporine A with or without 0.5 *μ*mol/L of VP16 as indicated for 24 h. Cell viability was examined by trypan blue staining. The graph chart represents the mean ± SD of three independent experiments. (D and E) EBV-T-LPDs cell lines, SNT8 (D) and SNT16 (E) were cultured with 2 *μ*mol/L of cyclosporine A with or without 10 nmol/L of doxorubicin as indicated for 24 h. Cell viability was examined by trypan blue staining. The graph chart represents the mean ± SD of three independent experiments. (F) PBMCs from EBV-T-LPDs patients (case CD4-1) were cultured in 10% FCS–RPMI containing IL-2 with 1 *μ*mol/L of cyclosporine A with or without 10 nmol/L of doxorubicin as indicated for 24 h. Cell viability was examined by trypan blue staining. The graph chart represents the mean ± SD of three independent experiments. EBV-T-LPDs, Epstein–Barr virus-positive T-cell lymphoproliferative diseases; PBMCs, peripheral blood mononuclear cells; FCS, fetal calf serum; IL-2, interleukin-2.

Next, we examined the effects of P-gp on chemoresistance of EBV-T-LPDs. Etoposide and doxorubicin, chemotherapeutic agents which are often used to treat lymphoid neoplasms, are substrates of P-gp 25–28. SNT8 and SNT16 cells were cultured with etoposide in the presence or absence of CsA. As shown in Figure4A and B, etoposide-induced cell death was enhanced by CsA in SNT8 and SNT16 cells, suggesting that P-gp suppressed etoposide-induced cell death in EBV-positive T cells. Then, we validated the results in patient cells. PBMCs of case CD4-1 were obtained and cultured with etoposide in the presence or absence of CsA. As shown in Figure4C, CsA enhanced etoposide-induced cell death. The similar results were obtained from the assay using doxorubicin. As shown in Figure4D–F, doxorubicin-induced cell death was enhanced by CsA in SNT8, SNT16, and CD4-1 cells. We also examined the effects of CsA on L-asp which was not a substrate of P-gp. As shown in Figure S1, CsA did not have significant effect on L-asp-induced cell death.

**Figure 5:**
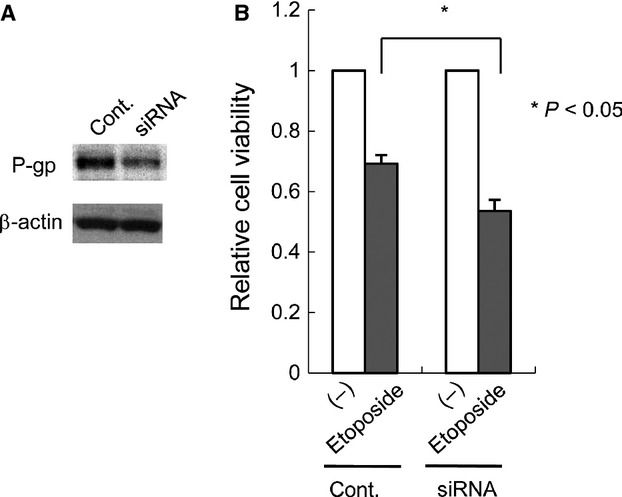
The effects of P-glycoprotein (P-gp) down regulation by siRNA on etoposide-induced cell death in an Epstein–Barr virus-positive T-cell lymphoproliferative diseases cell line. (A) SNT16 cells were transfected with 6 *μ*g of siRNA-*mdr1* or siRNA as described under Materials and Methods. After cultured for 2 days, cell lysates were prepared from an aliquot of cells and subjected to anti-P-gp immunoblotting, followed by reprobing with anti-*β* actin for loading control. (B) Transfected cells were treated with 2 *μ*mol/L etoposide for 24 h and subjected to the viability assay by trypan blue staining. The graph chart represents the mean ± SD of three independent experiments.

Next, we examined whether P-gp directly contributed to the resistance of EBV-T-LPDs. siRNA transfection resulted in decreased P-gp expression in SNT16 cells as shown in Figure5A. Etoposide-induced cell death occurred significantly more often in the transfected cells than in the control cells (Fig.5B). These results indicated that P-gp directly could induce chemoresistance of EBV-positive cells from EBV-T-LPDs.

### EBV enhanced functional expression of P-gp in T cells

**Figure 6:**
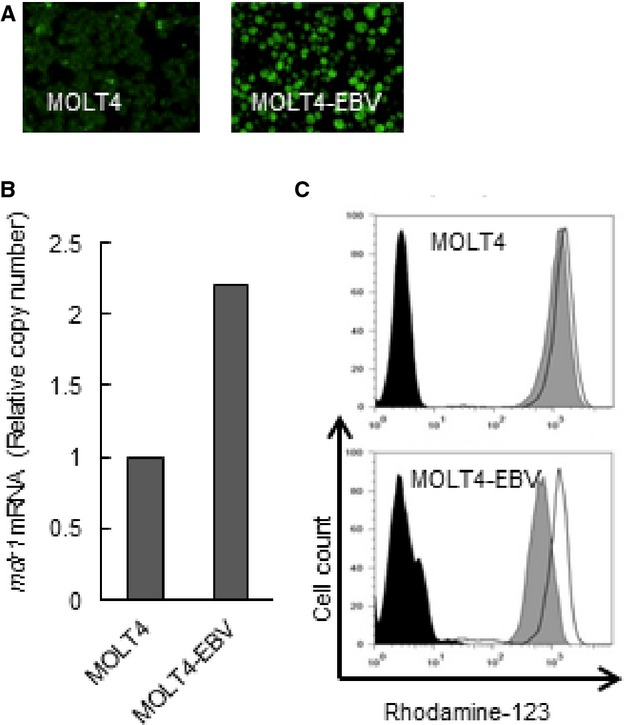
The effects of EBV infection on P-gp expression in MOLT4 cells. (A) Original (left), EBV-infected MOLT4 (right) were obtained for EBNA1 staining. Green fluorescent cells are EBNA1-positive. (B) RNA expression of *mdr1* was examined in the original and EBV-infected MOLT4 cells as described in the manuscript. Transcripts of *mdr1* and *GAPDH* of each cell line were quantitated by real-time RT-PCR. Relative copy number was obtained by normalizing the *mdr1* transcripts to those of *GAPDH*. (C) Function of P-gp in the original and EBV-infected MOLT4 cells was determined by Rhodamine 123 efflux assay as described in the manuscript. Cells were incubated with 500 ng/mL of Rhodamine-123 for 30 min at 37°C, then allowed to efflux the dye in dye-free 10% FCS-RPMI for 2 h at 37°C (gray, shaded histogram) or at 4°C (open histogram). After efflux, cells were analyzed using flow cytometer. The untreated cells were presented in black, shaded histogram. P-gp, P-glycoprotein; EBV, Epstein–Barr virus; EBNA, EBV nuclear antigen; *GAPDH*, glyceraldehyde-3-phosphate dehydrogenase; RT-PCR, reverse-transcriptase polymerase chain reaction; FCS, fetal calf serum.

To clarify whether EBV could directly induce P-gp expression, we finally performed EBV infection assay on MOLT4 cells. EBV infection was confirmed by the detection of EBV DNA and EBNA staining. The EBV DNA copy number of EBV-infected MOTL4 cells was 8.8 × 10^5^ copies/*μ*g DNA. EBV infection was verified by the presence of EBNA 1 protein expression. Most cells were positive for EBNA1 (Fig.6A). Then, we examined P-gp expression in EBV-infected MOLT4 cells. As shown in Figure6B, quantitative RT-PCR revealed that *mdr1* mRNA was enhanced in EBV-infected MOTL4 cells. In addition, EBV infection also upregulated Rhodamine efflux by MOLT4 cells (Fig.6C). These results indicated that EBV itself enhanced the functional expression of P-gp in T cells.

## Discussion

An optimal chemotherapy for EBV-T-LPDs has not been established. Immunochemotherapy consisting of CsA, etoposide, and prednisolone followed by chemotherapy has been the most commonly used EBV-T-LPDs treatment 5. Some patients respond to this treatment, and their disease becomes inactive; however, EBV-positive T cells cannot be eradicated in most cases. In this study, we demonstrated that EBV-positive T cells in EBV-T-LPDs expressed functional P-gp protein. We also detected that suppressing P-gp using siRNA and the P-gp inhibitor CsA enhanced etoposide or doxorubicin-induced cell death in these cells. These results indicated that P-gp may cause the resistance of EBV-T-LPDs to chemotherapies. It was reported that anticancer agents including doxorubicin induced P-gp expression 27,28. The enhancement of cell death by CsA in etoposide- or doxorubicin-treated SNT8 cells (Figs.4A and 5A) was relatively clear as compared with P-gp function in the cells (Fig.3C). Similar mechanisms may exist in SNT8.

The mechanism that regulates P-gp expression in EBV-T-LPDs has not been clarified to date. It has been reported that normal circulating lymphocytes including CD4-, 8-, 56-positive cells expressed P-gp 29. Since the tumor cells from EBV-T-LPDs are peripheral T cells in the PB, they can naturally express P-gp. In addition, we demonstrated that EBV infection directly enhanced functional P-gp expression in MOLT4 cells. However, we have not succeeded in *in vitro* EBV infection on peripheral blood T lymphocytes yet. Since EBV-positive tumor cells in EBV-T-LPDs are peripheral T lymphocytes, further investigation is ongoing in our laboratory to confirm the roles of EBV on the cells. P-gp expression could be induced by Akt 25,30, Erk 31, and JNK 32 signaling pathways. EBV-infected T cells reveals type 2 latent infection and the viral protein LMP1 is expressed in them. EBV-infected MOLT4 cells expressed *LMP1*, *LMP2A*, *LMP2B*, and *EBNA1* and were also considered latency type 2 15. As LMP1 induces Akt 33, Erk 34, and JNK 35 activation in EBV-infected cells, it may also mediate EBV-induced P-gp expression. Furthermore, P-gp expression is epigenetically regulated by methylation 36. Since LMP-1 induces H3K27me3 demethylase, KDM6B 37, it can be suggested that LMP1 induces hypomethylation of *mdr1* resulting in P-gp overexpression. Further research is needed to clarify the role of LMP1 for P-gp expression.

Our results showed that suppression of P-gp enhanced etoposide- and doxorubisin-induced cell death in EBV-T-LPDs cells. We also demonstrated that CsA worked as a P-gp inhibitor, and its concentration which enhanced cell death in patient's cells was within range for clinical use. In this point, immunochemotherapy (CsA, etoposide, and prednisolone), which has been suggested by Koyama et al., is reasonable 5. CsA and some P-gp inhibitors, such as verapamil, are available. Adding them to chemotherapeutic regimens is worth being considered. Furthermore, the regimens which are not influenced by P-gp should be suggested. For instance, L-asp, one of the reagents used to treat lymphoid neoplasms, is not a substrate of P-gp 38. P-gp expression in ENKL, another EBV-positive NK-LPD, was examined first by Yamaguchi and colleagues by immunohistochemistry. They found that tumor cells from nine of 10 ENKL patients were positive for P-gp 12. In accordance with the findings, ENKL revealed resistant for CHOP-like chemotherapy 6. Recently, Yamaguchi et al. suggested SMILE regimen mainly consisting of P-gp-independent reagents including L-asp, and reported its efficacy on ENKL 39. In addition, it was reported that chemotherapy consisting L-asp had also effect on ANKL 40. Since L-asp is effective against NK/T-cell neoplasia even as a single reagent 38,41, the efficacy of chemotherapy containing L-asp on EBV-T-LPD is expected.

In conclusion, EBV-infected T cells in EBV-T-LPDs express functional P-gp, which may be a cause of chemoresistance. Not only EBV-T-LPDs but also some T-cell lymphomas carry the EBV genome in their tumor cells. For these diseases, P-gp can be a molecular target to overcome the resistance. And treatment strategies consisting of P-gp-independent reagents should be considered to improve the therapeutic outcome.

## Supporting Information

**Figure S1.** The effects of P-glycoprotein inhibitor, cyclosporine A, on L-asparaginase-induced cell death in EBV-T-LPDs cells. (A and B) EBV-T-LPDs cell lines, SNT8 (A) and SNT16 (B) were cultured with 2 *μ*mol/L of cyclosporine A with or without 0.1 U of L-asparaginase as indicated for 24 h. Cell viability of each cell line was examined by trypan blue staining. The graph chart represents the mean ± SD of three independent experiments. (C) PBMCs from EBV-T-LPDs patients (case CD4-1) were cultured in 10% FCS–RPMI containing IL-2 with 1 *μ*mol/L of cyclosporine A with or without 0.1 U of L-asparaginase as indicated for 24 h. Cell viability was examined by trypan blue staining. The graph chart represents the mean ± SD of three independent experiments.

**Table S1.** The patients of EBV-negative diffuse large B-cell lymphoma examined in the study.
