# Supplementary figures and images for: P-glycoprotein is expressed and causes resistance to chemotherapy in EBV-positive T-cell lymphoproliferative diseases
Source: Cancer Med. 2015 Jul 8;4(10):1494–504. doi: 10.1002/cam4.494 (PMC4618620; doi:10.1002/cam4.494)

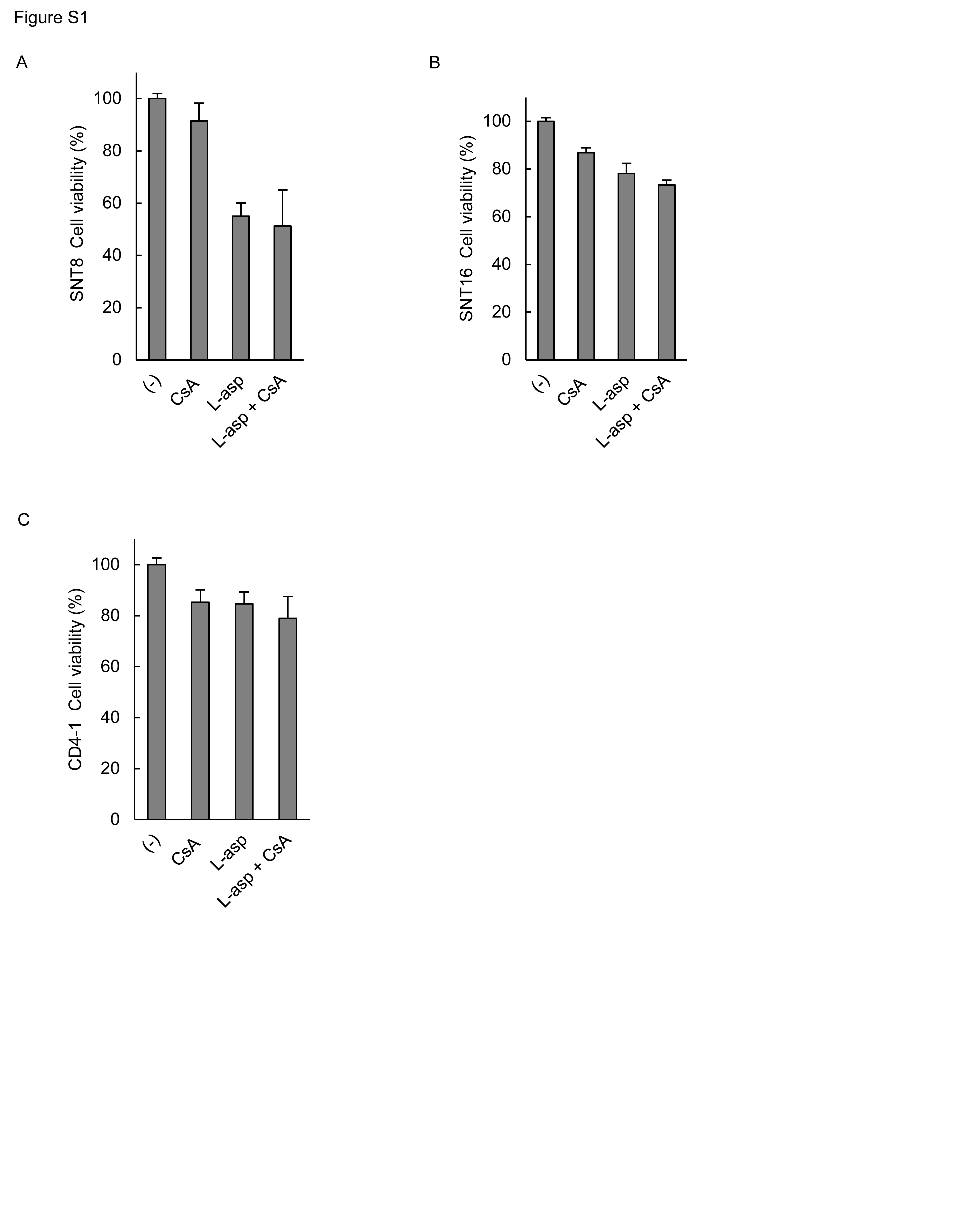

Supplement: Supplementary file 1 [file cam40004-1494-sd1.jpg]
