# Supplementary material for: P-glycoprotein is expressed and causes resistance to chemotherapy in EBV-positive T-cell lymphoproliferative diseases
Source: Cancer Med. 2015 Jul 8;4(10):1494–504. doi: 10.1002/cam4.494 (PMC4618620; doi:10.1002/cam4.494)
Supplement: Supplementary file 2 [file cam40004-1494-sd2.docx]

Supplementary Table 1 The patients of EBV-negative diffuse large B-cell lymphoma examined in the study

| Case | Gender | Age | Sites of involvement | treatment | Response　for CHOP |
| --- | --- | --- | --- | --- | --- |
| DLBCL-1 | F | 64 | Cervical LNs | CHOP | CR |
| DLBCL-2 | M | 67 | Systemic LNs | CHOP | CR |
| DLBCL-3 | F | 72 | Systemic LNs | CHOP | CR |

DLBCL : diffuse large B-cell lymphoma, M : Male, F: Female , LN : lymph node, CR: complete response,

CHOP consists of cyclophosphamide, doxorubicin, vincristine, and prednisolone
